# Dietary α-linolenic acid-rich flaxseed oil prevents against alcoholic hepatic steatosis *via* ameliorating lipid homeostasis at adipose tissue-liver axis in mice

**DOI:** 10.1038/srep26826

**Published:** 2016-05-25

**Authors:** Meng Wang, Xiao-Jing Zhang, Kun Feng, Chengwei He, Peng Li, Yuan-Jia Hu, Huanxing Su, Jian-Bo Wan

**Affiliations:** 1 State Key Laboratory of Quality Research in Chinese Medicine, Institute of Chinese Medical Sciences, University of Macau, Macao; 2 Department of Bioengineering, Zunyi Medical College, Zhuhai Campus, Zhuhai, Guangdong, China

## Abstract

Low levels of n-3 polyunsaturated fatty acids (PUFAs) in serum and liver tissue biopsies are the common characteristics in patients with alcoholic liver disease. The α-linolenic acid (ALA) is a plant-derived n-3 PUFA and is rich in flaxseed oil. However, the impact of ALA on alcoholic fatty liver is largely unknown. In this study, we assessed the potential protective effects of ALA-rich flaxseed oil (FO) on ethanol-induced hepatic steatosis and observed that dietary FO supplementation effectively attenuated the ethanol-induced hepatic lipid accumulation in mice. Ethanol exposure stimulated adipose lipolysis but reduced fatty acid/lipid uptake, which were normalized by FO. Our investigations into the corresponding mechanisms demonstrated that the ameliorating effect of FO might be associated with the lower endoplasmic reticulum stress and normalized lipid metabolism in adipose tissue. In the liver, alcohol exposure stimulated hepatic fatty acid uptake and triglyceride synthesis, which were attenuated by FO. Additionally, dietary FO upregulated plasma adiponectin concentration, hepatic adiponectin receptor 2 expression, and the activation of hepatic adenosine monophosphate-activated protein kinase. Collectively, dietary FO protects against alcoholic hepatic steatosis by improving lipid homeostasis at the adipose tissue-liver axis, suggesting that dietary ALA-rich flaxseed oil might be a promising approach for prevention of alcoholic fatty liver.

Consistently excessive alcohol intake leads to alcoholic liver disease (ALD), a major cause of morbidity and mortality worldwide among people who abuse alcohol^1^. ALD encompasses a histological spectrum of liver damage ranging from simple steatosis (fatty liver) to hepatitis, progressive fibrosis, and cirrhosis^2^. Hepatic steatosis, characterized by lipid accumulation in the cytoplasm of hepatocytes, has been thought to be the earliest response to alcohol intake and a reversible pathological condition^2^. Thus, reducing alcohol-induced hepatic fat accumulation may block or delay the progression of steatosis to more advanced stages of ALD. Multiple mechanisms contribute to the pathogenesis of alcoholic hepatic steatosis, including increased hepatic *de novo* lipogenesis, impaired mitochondrial fatty acid β-oxidation and decreased very low-density lipoprotein (VLDL) export^2^. Recently, increasing evidence has suggested that extra-hepatic factors, such as adipose-derived fatty acids and adiponectin, critically modulate hepatic lipid metabolism^3^,^4^.

White adipose tissue (WAT), a major organ for fat storage and adipokines secretion, plays a key role in regulating whole-body lipid homeostasis. Serving as an energy reservoir, WAT stores excess energy in the form of triglycerides (TGs) in a positive energy condition and mobilizes free fatty acids in a negative energy condition^5^. Dysfunctions of fat storage in adipose tissue may increase adipocyte lipolysis, subsequently causing excessive adipose-derived fatty acid influx into the liver, eventually resulting in hepatic steatosis^3^,^5^. Numerous clinical and animal studies have demonstrated that alcoholic hepatic steatosis is accompanied by reduced adipose mass^6^ and increased fatty acid uptake by hepatocytes^7^. As an endocrine organ, adipose tissue also secretes several adipokines, such as adiponectin that modulates hepatic lipid homeostasis towards a reduction of lipid content^5^. Considerable evidence has indicated that lower adiponectin production in adipose tissue and the impaired expression of adiponectin receptors in liver are responsible for the pathogenesis of alcoholic hepatic steatosis^8^. Circulating adiponectin achieves its bioactivity by binding to specific membrane-bound receptors, especially adiponectin receptor 2 (ADIPOR2), which is predominantly expressed in the liver. The activated adiponectin signaling leads to activation of the adenosine monophosphate-activated protein kinase (AMPK) pathway, which modulates hepatic lipid metabolism by simultaneously inhibiting *de novo* lipogenesis and stimulating fatty acid β-oxidation^4^. Therefore, healthy adipose tissue is essential for maintaining lipid homeostasis at the adipose tissue-liver axis.

Accumulating clinical evidence has revealed that a low levels of omega-3 (n-3) polyunsaturated fatty acids (PUFAs), including α-linolenic acid (ALA, 18:3 n-3), in serum and liver tissue biopsies is a common characteristic of patients with alcoholic and non-alcoholic liver disease^9^,^10^. It is possible that the supplementation of n-3 PUFAs could attenuate hepatic steatosis induced by alcohol exposure. An increasing number of studies have addressed the role of fish oil or long chain n-3 PUFAs (*e.g*., eicosapentaenoic acid (EPA, 20:5 n-3), docosahexaenoic acid (DHA, 22:6 n-3)) in alcoholic hepatic steatosis^11^,^12^,^13^,^14^,^15^,^16^. However, the results of these studies have been inconsistent. Recently, several studies have reported that fish oil or long chain n-3 PUFAs protect against fatty liver induced by alcohol^12^,^13^,^14^. Still, several studies have shown that fish oil promotes the pathogenesis of ethanol-induced hepatic steatosis and liver injury^15^,^16^. ALA is a plant-derived n-3 PUFAs that is more available and economical compared with EPA and DHA. However, its impact on ethanol-induced hepatic steatosis has not been addressed. A simple mouse model of chronic ethanol feeding plus a single binge can synergistically induce fatty liver and mimics acute-on-chronic alcoholic liver injury in humans^17^. Therefore, the aims of this study were to evaluate the possible protective effects of dietary ALA-rich flaxseed oil against alcoholic hepatic steatosis in a mouse model of chronic-plus-single-binge ethanol feeding, and to investigate its impacts on lipid homeostasis at the adipose tissue-liver axis.

## Results

### Routine parameters

As shown in Table 1, there was no significant difference in final body weight between four groups, whereas the chronic ethanol feeding plus a single binge alcohol feeding significantly elevated liver weights and the ratio of liver-to-body weight regardless of dietary fat. Compared with the alcohol-fed with corn oil (AF/CO) group, the ratio of liver-to-body weight in the alcohol-fed with flaxseed oil (AF/FO) group was notably reduced after ethanol administration. Meanwhile, alcohol exposure significantly reduced the epididymal adipose tissue (eWAT) mass and the eWAT-to- body weight ratio regardless of dietary fat, those in the AF/FO were considered to be within the normal range. In addition, the plasma aspartate aminotransferase (AST) and alanine aminotransferase (ALT) levels in the AF/CO group were significantly elevated by 2.3-fold (73.6 ± 11.7 vs. 22.9 ± 5.1 U/L) and 2.4-fold (50.8 ± 15.1 vs.14.9 ± 4.3 U/L), respectively, over those in their pair-fed mice, which were effectively suppressed by dietary FO administration. Alcohol-fed mice had a significantly lower plasma glucose concentration compared with their pair-fed controls, while neither alcohol nor fat affected the plasma insulin level. To determine whether this ameliorating effect of FO is associated with the absorption of alcohol in the digestive organs, the circulating alcohol concentrations were measured (Table 1). After chronic-plus-single-binge ethanol feeding, the plasma alcohol concentrations were elevated (8- to 10-fold) in both CO and FO groups, but FO did not significantly change the plasma ethanol level after alcohol exposure.

### Dietary FO alters fatty acid composition in liver and adipose tissues

Dietary FO greatly altered the fatty acid composition in the liver and adipose tissues. Compared with the CO-fed mice, the FO-fed mice had the significantly higher levels of ALA, EPA, docosapentaenoic acid (DPA, 22:5 n-3), and total n-3 PUFAs, and lower contents of linoleic acid (LA, 18:2 n-6), arachidonic acid (AA, 20:4 n-6), and total n-6 PUFAs in liver samples (Table 2). Interestingly, dietary FO had no significant effect on the hepatic DHA level, which was consistent with previous studies^18^. Similar alterations in the fatty acid profile were also observed in adipose tissue (see Supplementary Table S1).

### Dietary FO attenuated alcohol-induced hepatic lipid accumulation

**Figure 1:**
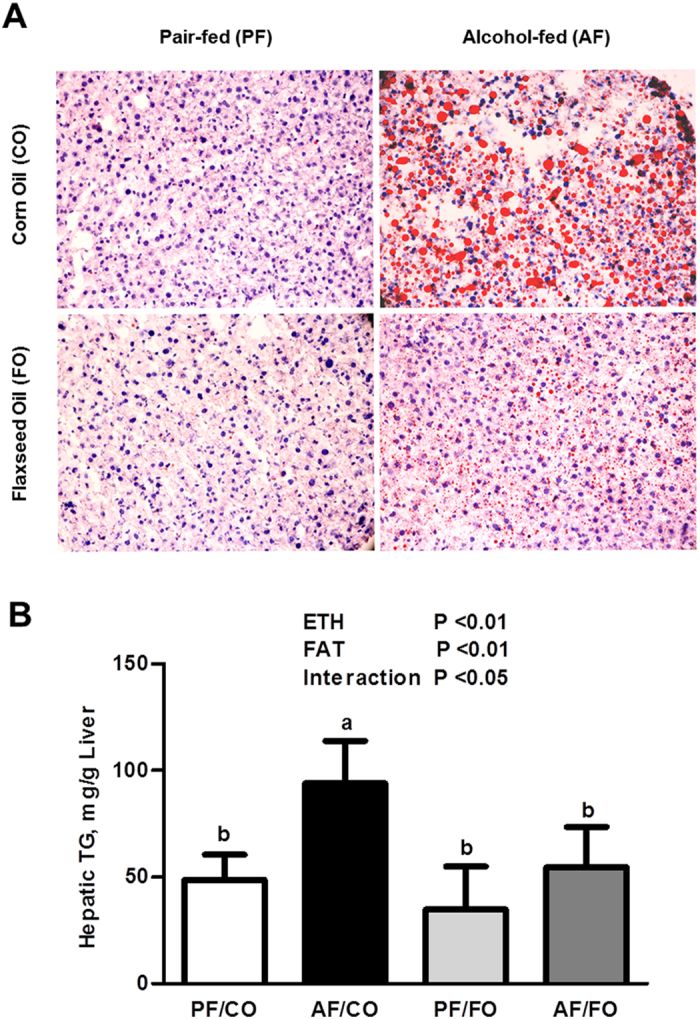
Dietary flaxseed oil alleviates hepatic lipid accumulation in alcohol-fed mice. Representative Oil Red O staining (200× magnification) of the liver (**A**) and the concentrations of hepatic triglycerides (**B**). Values represent the means ± SD (n = 8–10). Labeled means without a common letter are significantly different (*P* < 0.05). AF/CO, alcohol-fed with corn oil; AF/FO, alcohol-fed with flaxseed oil; ETH, ethanol; PF/CO, pair-fed with corn oil; PF/FO, pair-fed with flaxseed oil.

After alcohol exposure, mice in the alcohol-fed groups (*i.e.*, AF/CO and AF/FO) exhibited the greater microvesicular steatosis in liver sections compared with their pair-fed groups, as illustrated by Oil Red O staining (Fig. 1A). However, the lipid droplets in the livers of the AF/FO group were much fewer in number and smaller than those of the AF/CO group. Quantitative TG measurement confirmed the histological results by demonstrating that alcohol feeding with CO greatly elevated the hepatic TG level in mice by 92.9% compared with the pair-fed group, and this elevation was significantly alleviated by FO supplementation (Fig. 1B). These data clearly indicated that FO could effectively protect against ethanol-induced hepatic steatosis.

### Dietary FO ameliorates adipose tissue lipolysis stimulated by alcohol exposure

**Figure 2:**
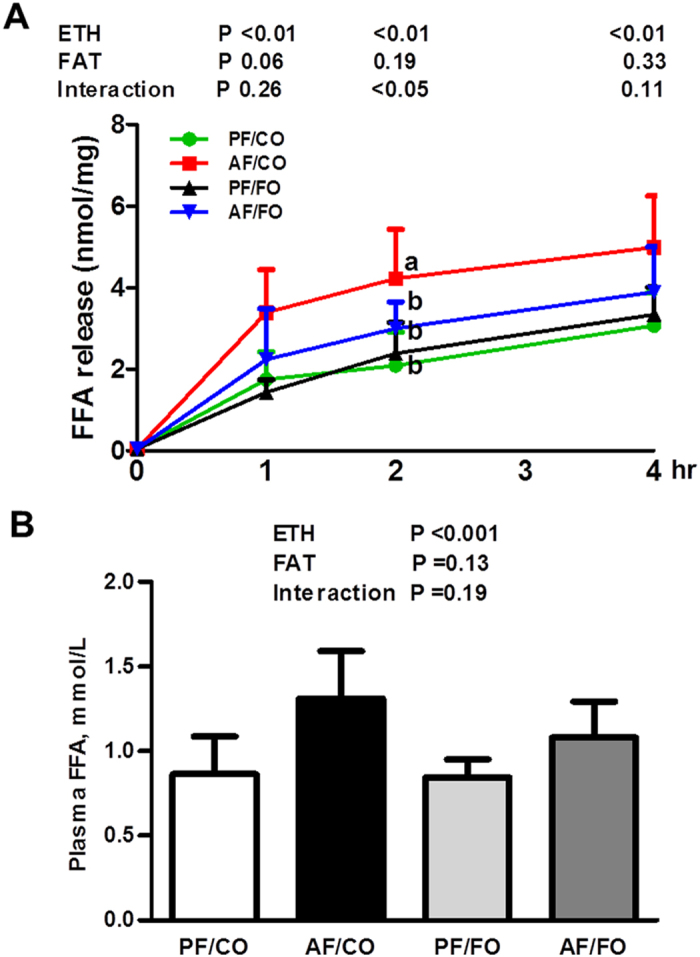
Dietary flaxseed oil alleviates adipose lipolysis (**A**) and the plasma free fatty acid concentration (**B**) in alcohol-fed mice. Adipose lipolysis was estimated by measuring the free fatty acid released from epididymal WAT explants *ex vivo*. Values represent the means ± SD (n = 8–10). Labeled means without a common letter are significantly different (*P* < 0.05). AF/CO, alcohol-fed with corn oil; AF/FO, alcohol-fed with flaxseed oil; ETH, ethanol; FFA, free fatty acids; PF/CO, pair-fed with corn oil; PF/FO, pair-fed with flaxseed oil.

As shown in Fig. 2A, freshly isolated epididymal WAT from the AF/CO group showed a significantly higher capacity for free fatty acid (FFA) release during a 4-h incubation compared with that from pair-fed mice. Dietary FO abolished the higher FFA release from epididymal WAT induced by ethanol intake. These results were consistent with the alterations in plasma FFA level; plasma FFA concentration was significantly higher in the AF/CO, whereas, the mice in the AF/FO group were resistant to alcohol-induced plasma FFA elevation (Fig. 2B).

**Figure 3:**
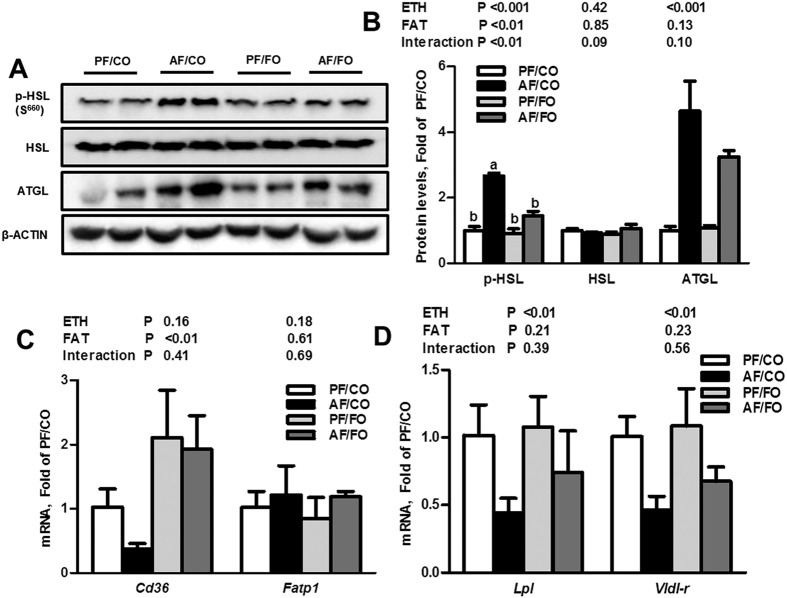
Dietary flaxseed oil improves the dysfunctional lipid metabolism of adipose tissue in alcohol-fed mice. Immunoblot analysis of p-HSL, HSL, and ATGL proteins related to adipose lipolysis in epididymal adipose tissue (**A**) and their results of the densitometric analysis (**B**). qPCR analysis of genes related to fatty acid transport (**C**) and VLDL uptake (**D**) in epididymal adipose tissue. Values represent the means ± SD (n = 3–4); Labeled means without a common letter are significantly different (*P* < 0.05). AF/CO, alcohol-fed with corn oil; AF/FO, alcohol-fed with flaxseed oil; ETH, ethanol; PF/CO, pair-fed with corn oil; PF/FO, pair-fed with flaxseed oil.

**Figure 4:**
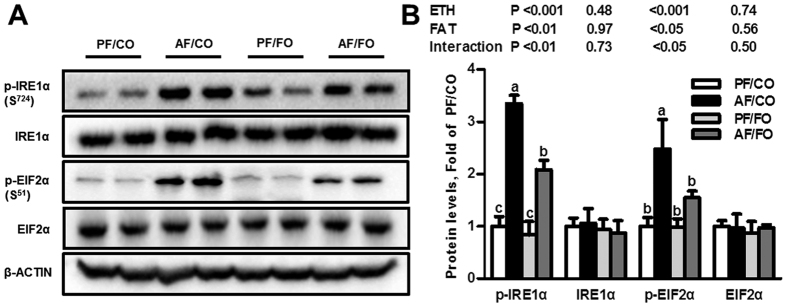
Dietary flaxseed oil ameliorates endoplasmic reticulum stress in the epididymal adipose tissue of alcohol-fed mice. Representative Western blot images of p-IRE1α, IRE1α, p-EIF2α, and EIF2α proteins (**A**) and the densitometry analysis results (**B**). Values represent the means ± SD (n = 3–4). Labeled means without a common letter are significantly different (*P* < 0.05). AF/CO, alcohol-fed with corn oil; AF/FO, alcohol-fed with flaxseed oil; ETH, ethanol; PF/CO, pair-fed with corn oil; PF/FO, pair-fed with flaxseed oil.

Ethanol feeding with CO as the fat source significantly up-regulated the protein levels of adipose triglyceride lipase (ATGL) and phosphor-hormone-sensitive lipase (p-HSL, Ser^660^) in epididymal WAT by 3.6- and 1.7-fold, respectively, which was significantly inhibited by dietary FO. The total HSL protein level was not affected (Fig. 3A,B). Fatty acid transport and lipid uptake are important functions of adipose fat storage. Ethanol feeding with CO significantly down-regulated the genes involved in VLDL uptake, *i.e.*, lipoprotein lipase (*Lpl*) and very low-density lipoprotein receptor (*Vldl-r*), and the expression of fatty acid translocase (*Cd36*) in adipose tissue, but the mRNA expression of a gene involved in fatty acid transport (fatty acid transporter protein 1 (*Fatp1*)) was not significantly influenced by alcohol administration (Fig. 3C,D). However, only the *Cd36* expression was significantly elevated by FO feeding. Our data demonstrated that dietary FO improved ethanol-induced WAT dysfunction, which is associated with decreased alcohol-stimulated WAT lipolysis and normalization of the function of adipose fat storage. Additionally, alcohol exposure with CO significantly up-regulated the protein levels of the phosphorylated inositol-requiring enzyme 1α (p-IRE1α) and the phosphorylated eukaryotic translational initiation factor 2α (p-EIF2α), compared with the pair-fed with corn oil (PF/CO) group, which were partially inhibited by dietary FO, whereas IRE1α and EIF2α were not affected (Fig. 4).

### Dietary FO improved alcohol-induced lipid dyshomeostasis in the liver

**Figure 5:**
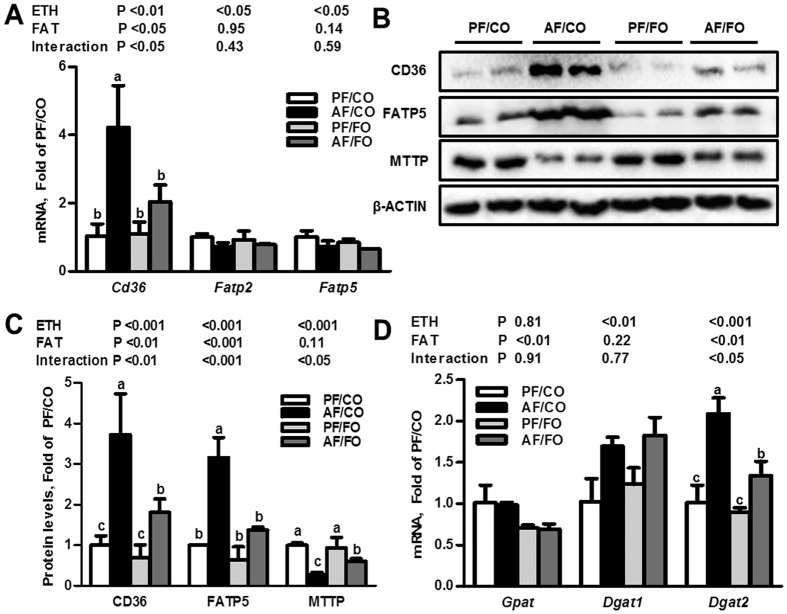
Dietary flaxseed oil improves the dysfunctional lipid metabolism in the liver of alcohol-fed mice. qPCR analysis of liver genes involved in fatty acid uptake (**A**) and triglyceride synthesis (**B**). Immunoblot analysis of genes involved in fatty acid uptake and VLDL export in the liver (**C**) and their results of the densitometric analysis (**D**). Values represent the means ± SD (n = 3–4). Labeled means without a common letter are significantly different (*P* < 0.05). AF/CO, alcohol-fed with corn oil; AF/FO, alcohol-fed with flaxseed oil; ETH, ethanol; PF/CO, pair-fed with corn oil; PF/FO, pair-fed with flaxseed oil.

Alcohol exposure with CO significantly elevated the transcriptional level of *Cd36* (Fig. 5A), but not *Fatp2* and *Fatp5*, and also up-regulated expressions of CD36 and FATP5 protein compared with the PF/CO group (Fig. 5B,C). All of these up-regulating effects of ethanol were normalized by dietary FO.

As shown in Fig. 5D, the mRNA level of glycerol-3-phosphate acyltransferase (*Gpat*) was not significantly affected by ethanol intake regardless of dietary fat. Unlike *Gpat*, ethanol exposure elevated the hepatic mRNA levels of diacylglycerol acyltransferase 1 (*Dgat1*) and *Dgat2* in both CO-fed and FO-fed mice; however, dietary FO significantly reduced the expression of *Dgat2* but not *Dgat1* compared with the AF/CO group. Additionally, alcohol exposure with CO also down-regulated the protein expression of microsomal triglyceride transfer protein (MTTP), an essential lipid transfer protein for the assembly and secretion of VLDL, which was protected after the replacement of CO with FO (Fig. 5B,C).

### Dietary FO improved the inhibitory effect of ethanol on adiponectin secretion and signaling

**Figure 6:**
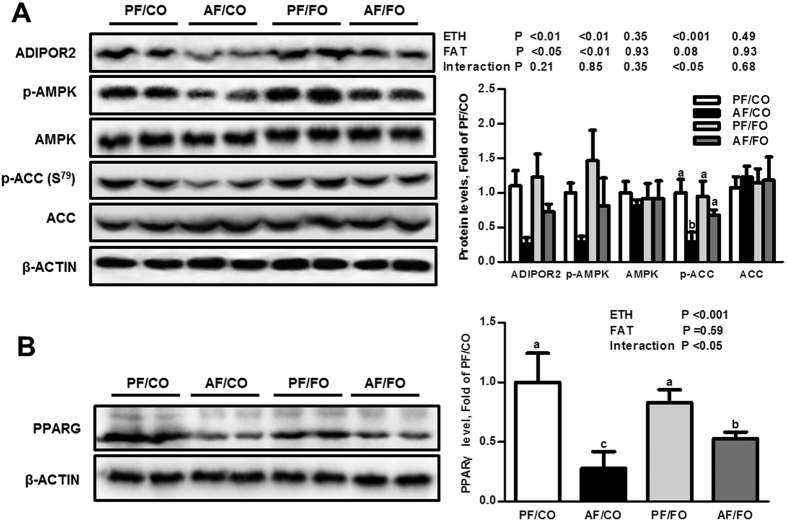
Dietary flaxseed oil improves the ethanol inhibitory effect on adiponectin signaling in alcohol-fed mice. Immunoblot analysis of ADIPOR2, ACC, AMPK, p-AMPK (Thr^172^), and p-ACC (Ser^79^) proteins in the liver (**A**) and PPARG in epididymal adipose tissue (**B**). Right panels show the densitometric analysis results. Values represent the means ± SD (n = 3–4). Labeled means without a common letter are significantly different (*P* < 0.05). AF/CO, alcohol-fed with corn oil; AF/FO, alcohol-fed with flaxseed oil; ETH, ethanol; PF/CO, pair-fed with corn oil; PF/FO, pair-fed with flaxseed oil.

Alcohol exposure significantly reduced the circulating adiponectin concentration in both the CO- and FO-fed mice, whereas, mice in the AF/FO showed normal plasma adiponectin level (Table 1). Regardless of dietary fat, alcohol exposure significantly down-regulated hepatic protein levels of ADIPOR2 (an adiponectin receptor expressed in the liver), phosphorylated AMPK (p-AMPK), but not total AMPK, whereas the expression levels of ADIPOR2 and p-AMPK in the AF/FO were comparable to those in the PF/CO (Fig. 6A). Alcohol exposure also significantly down-regulated hepatic protein expression of phosphorylated acetyl-CoA carboxylase (p-ACC) in CO-fed mice, but not in FO-fed mice (Fig. 6A). Additionally, alcohol exposure with CO also reduced the protein expression of peroxisome proliferator-activated receptor-γ (PPARG) compared with the PF/CO, which was improved by dietary FO (Fig. 6B).

## Discussion

**Figure 7:**
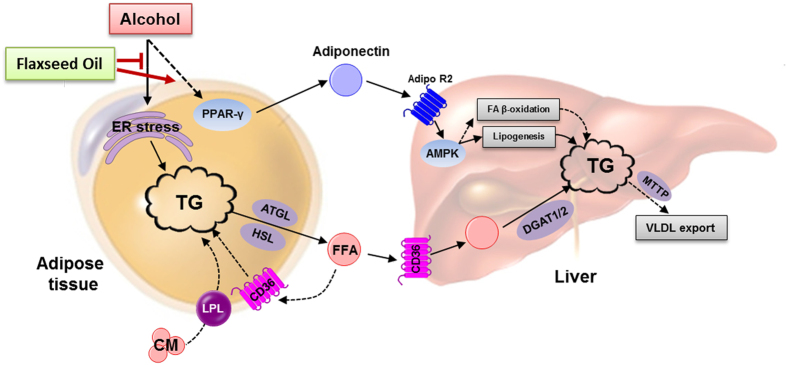
Schematic diagram of the potential mechanisms that underlie the protective effect of α-linolenic acid-rich flaxseed oil against alcoholic hepatic steatosis in mice. ADIPOR2, adiponectin receptor 2; AMPK, adenosine monophosphate-activated protein kinase; ATGL, adipose triglyceride lipase; CM, chylomicrons; DGAT, diacylglycerol acyltransferase; ER, endoplasmic reticulum; FFA, free fatty acid; GPAT, glycerol-3-phosphate acyltransferase; HSL, hormone-sensitive lipase; IRE1α, inositol-requiring enzyme 1α; LPL, lipoprotein lipase; MTTP, microsomal triglyceride transfer protein; PPARG, peroxisome proliferator-activated receptor-γ; TG, triglyceride; VLDL, very low-density lipoprotein; VLDL-r, very low-density lipoprotein receptor.

Despite the economic burden of ALD and its serious impacts on public health, few satisfactory advances have been made in the management of ALD, except abstinence from alcohol^2^. This study demonstrated that dietary ALA-rich flaxseed oil alleviated alcohol-induced hepatic steatosis by improving adipose function and lipid homeostasis at the adipose tissue-liver axis, as illustrated in Fig. 7. Dietary FO ameliorated ethanol-stimulated adipose lipolysis by attenuating endoplasmic reticulum (ER) stress, leading to a reduced fatty acid influx to the liver. Dietary FO also promoted adipose adiponectin production by up-regulating PPARG expression in adipose tissue, thereby, activating AMPK in the liver. Indeed, ALA serves as a precursor for the synthesis of long-chain n-3 polyunsaturated fatty acids, such as EPA and DHA. Accumulating clinical and animal studies have demonstrated that dietary supplementation of ALA significantly raised the EPA level and ALA levels in blood and tissues but had no significant effect on the DHA level^18^, which was consistent with our analysis of the fatty acid composition of the liver (Table 2) and adipose tissues (see Supplementary Table S1). Thus, this protective effect of dietary FO may be attributed to ALA itself and its conversion to EPA.

Hepatic steatosis is the earliest and most common response of the liver to either acute or chronic alcohol exposure. Overburdened lipids in the cytoplasm of the hepatocytes greatly influence cellular function and cause the hepatocytes to become susceptible to hepatotoxins. Therefore, suppressing hepatic lipid accumulation during alcohol exposure may block further liver damage. Our data showed that dietary FO markedly attenuated hepatic steatosis induced by chronic-plus-single-binge ethanol feeding, as indicated by Oil Red O staining and hepatic TG quantification. Recently, an increasing number of studies have demonstrated that adipose dysfunction plays a critical role in the pathogenesis of alcoholic steatosis^3^,^19^. Our results showed that alcohol exposure significantly reduced the epididymal WAT weight and its ratio to the body weight. Lipid homeostasis in WAT depends on adipose lipolysis and fatty acid/fat uptake, which changes the fat mass^20^. Adipocytes take up TGs hydrolyzed from circulating VLDL and/or chylomicrons (CMs), and release fatty acids by adipose lipolysis. Thus, adipose mass loss may be caused by either reduced TG uptake or increased adipose lipolysis. Numerous previous studies have demonstrated that chronic alcohol exposure not only stimulates the TG degradation in WAT but also suppresses lipid/fatty acid uptake^3^,^5^, which was consistent with our findings. This study also demonstrated that dietary FO ameliorated adipose lipid dyshomeostasis by normalizing the ethanol-impaired expression of adipose genes related to lipid metabolisms. ATGL and HSL are key rate-limiting hydrolases responsible for adipose lipolysis^21^. Dietary FO normalized ethanol-up-regulated expression of ATGL and phosphorylated HSL. CD36, a key fatty acid transporter, is involved in regulating fatty acid uptake across the plasma membrane in both adipose tissue and the liver. LPL is the key enzyme that is responsible for the hydrolysis of TG in TG-rich lipoproteins (*e.g.*, CMs and VLDL). In addition, LPL and Vldl-r are related to lipid uptake in adipose tissue^22^. Alcohol exposure down-regulated mRNA expression of *Cd36*, *Lpl* and *Vldl-r*, but only *CD36* expression was significantly elevated by FO feeding after alcohol exposure. Although adipose lipolysis is tightly modulated by several hormones, such as catecholamines and insulin^5^,^19^, chronic alcohol exposure did not affect the circulating levels of the positive regulators adrenaline and nonadrenaline or the negative regulator insulin^3^. Our results also indicated that dietary FO or alcohol did not change plasma insulin levels (Table 1). Instead, ethanol feeding suppressed β-adrenergic receptor-stimulated lipolysis^23^. It is therefore likely that there are other causes of ethanol-stimulated adipose lipolysis. One of these causes appears to be ER stress. Recent studies indicated that ER stress induced lipolysis in adipose tissue, which contributed to increases in circulating FFAs and fatty infiltration into other organs, including the liver^24^. The ER is an important organelle for protein synthesis and folding, lipid synthesis, and nascent lipid droplet formation. The lipolytic effect of ER stress occurred with elevated cyclic adenosine monophosphate (cAMP) production and protein kinase A (PKA) activity, leading to increased HSL phosphorylation and the resultant lipolysis in adipose tissue^24^. ER stress or dysfunction could initiate the unfolded protein response (UPR) *via* the activation of three transmembrane receptors in the ER membrane; *i.e.*, IRE1α, PRKR-like endoplasmic reticulum kinase (PERK), and the activating transcription factor 6 (ATF6). Thus, the proteins level of IRE1α and EIF2α, a PERK downstream protein, were examined by immunoblot analysis. Our results indicated that dietary FO attenuated ethanol-induced ER stress by partially down-regulating the protein expression of p-IRE1α and p-EIF2α elevated by alcohol exposure. Those effects may contribute to the ameliorating effects on WAT lipolysis.

As an endocrine organ, adipose tissue secretes several bioactive adipokines, including adiponectin. Adiponectin, a major adipose-derived adipokines that critically regulate lipid metabolism in the liver, plays a vital role in alcoholic fatty liver^25^. The reduced adiponectin production in adipose tissue, the decreased circulating adiponectin level and the impaired expression of ADIPORs in the liver are associated with the progression of alcoholic hepatic steatosis in several rodent models^4^,^26^. More importantly, the administration of full-length recombinant adiponectin attenuated alcoholic fatty liver and inflammation in mice^26^. The function of adiponectin in hepatic lipid metabolism is largely mediated by AMPK signaling in the liver, leading to enhanced fatty acid β-oxidation and reduced *de novo* lipogenesis. However, the effect of alcohol on adiponectin production remains controversial. Several animal and clinical studies have demonstrated that the circulating adiponectin level was positively correlated with alcohol consumption and liver injury^27^,^28^ but decreased after abstinence from alcohol^28^. This study demonstrated that chronic feeding plus a single binge ethanol feeding reduced plasma adiponectin concentration and down-regulated the expression of ADIPOR2 in the liver, thereby suppressing the activation of AMPK, which could be normalized by dietary FO. Adiponectin is thought to be a downstream effector or mediator of PPARG, and rosiglitazone, a PPARG agonist, stimulated adipose adiponectin production in alcohol-fed mice^5^. Our data indicated that dietary FO also up-regulated the lowered protein expression of PPARG in alcohol-fed mice.

In conclusion, this study demonstrated that dietary α-linolenic acid-rich flaxseed oil attenuated hepatic steatosis induced by chronic-plus-single-binge ethanol feeding by improving lipid homeostasis at the adipose tissue-liver axis. Restoration of adipose function by dietary FO was associated with suppressing ethanol-stimulated adipose lipolysis and increasing the impaired adiponectin production in adipose tissue. Dietary FO protected alcoholic fatty liver by decreasing the hepatic fatty acid uptake, increasing VLDL export, and activating the adiponectin-mediated AMPK. Our findings also suggested that dietary α-linolenic acid-rich flaxseed oil may be a promising approach for the prevention of alcoholic fatty liver.

## Materials and Methods

### Animals and treatments

Male C57BL/6 mice (8–10 wk old) were obtained from the Laboratory Animal Services Center, The Chinese University of Hong Kong (Hong Kong, China). A simple mouse model of alcoholic hepatic steatosis was induced by chronic ethanol feeding plus a single binge ethanol feeding^17^. Briefly, after a one-week period of acclimation to the control liquid diet, the mice were fed the modified Lieber-DeCarli liquid diets containing ethanol (alcohol-fed, AF, n = 10) or isocaloric maltose dextrin as the control (pair-fed, PF, n = 10) for 10 days. Mice in the AF groups were fed a modified Lieber-DeCarli alcohol liquid diet with an energy composition of 18% protein, 19% carbohydrate, 35% fat and 28% ethanol (TROPHIC Animal Feed High-tech Co., Ltd. Nantong, China), whereas animals in the PF groups were fed the Lieber-DeCarli control diet, in which maltose-dextrin (carbohydrate) isocalorically replaced ethanol. To examine whether flaxseed oil regulates alcoholic hepatic steatosis, 35% of the total calories were provided by either corn oil (rich in n-6 PUFAs) or flaxseed oil (rich in n-3 PUFAs). The component of the liquid diets and the fatty acid composition of dietary fats are shown in Supplementary Tables S2 and S3, respectively. Thus, there were four experimental groups: (*i*) pair-fed with corn oil (PF/CO), (*ii*) alcohol-fed with corn oil (AF/CO), (*iii*) pair-fed with flaxseed oil (PF/FO), and (*iv*) alcohol-fed with flaxseed oil (AF/FO). Group *i* and group *iii* were the pair-fed controls for group *ii* and group *iv*, respectively. The liquid diets were freshly prepared from powder daily according to the manufacturer’s instruction. Food intake of the alcohol-fed mice and average daily volume per mouse was monitored and calculated. The calculated volume was used to adjust the amount of control liquid diet given to pair-fed mice, so that alcohol- and pair-fed mice consume the equal amounts of diet. On day 11, the mice were gavaged with a single dose of 31.5% (*v/v*) ethanol (5 g/kg BW) or isocaloric maltose dextrin solution. After fasting for 9 h, the mice were euthanized, and blood samples were immediately collected for biochemical analysis. The entire liver and epididymal fat were immediately excised, and a portion of tissues from the same lobe of the liver in each mouse was embedded in OCT (frozen tissue matrix) for histological analysis. Epididymal fat and the remaining liver tissue were stored at −80 °C until analysis. All animal experiments were conducted according to the procedures approved by the Animal Ethics Committee (Approval No. AEC-13-002-1), Institute of Chinese Medical Sciences, University of Macau.

### Analysis of fatty acid composition in liver and adipose tissues

The fatty acid compositions of liver and adipose tissues were determined by gas chromatography-mass spectrometry (GC-MS) as described previously^29^. The relative content of each fatty acid in the tissues was quantified by normalization of the peak areas as the percentages of total fatty acids in the GC-MS chromatogram.

### Measurements of blood parameters

Plasma alcohol concentrations were measured using an Ethanol Assay Kit (Sigma-Aldrich, St. Louis, MO, USA). Plasma enzyme activities of AST and ALT were colorimetrically determined by the corresponding commercial assay kits (Nanjing Jiancheng Bioengineering Institute, Nanjing, China), according to the manufacturer’s instructions. Plasma levels of free fatty acid, glucose, and insulin were measured by a Free Fatty Acid Quantification Colorimetric Kit (BioVision, Milpitas, CA, USA), a glucose assay kit (Nanjing Jiancheng Bioengineering Institute, Nanjing, China), and a Mouse Insulin ELISA kit (Westang Biological Technology Co., Ltd, Shanghai, China), respectively, according to the manufacturers’ instructions.

### Determination of hepatic lipid accumulation

Hepatic lipid accumulation was qualitatively assessed by Oil Red O staining as previously described^30^. The hepatic TG content was also quantitatively determined using a triglyceride quantification kit (Beijing BHKT Clinical Reagent Co., Ltd, Beijing, China) following the manufacturer’s protocol. TG concentrations were expressed relative to wet liver weight (mg/g).

### Fatty acid release from *ex vivo* WAT cultures

Lipolysis of adipose tissue was measured as FFA released into the WAT culture medium *ex vivo* at different time intervals as described previously^30^. The released FFA levels in the culture medium at 0 h, 1 h, 2 h and 4 h were measured using a Free Fatty Acid Quantification Colorimetric Kit (Biovision, Milpitas, CA) according to the manufacturer’s instruction.

### Measurements of plasma adiponectin

Plasma adiponectin was determined by a mouse adiponectin/Acrp30 Quantikine ELISA kit (R&D Systems, Minneapolis, MN, USA) according to the manufacturer’s protocol. Plasma samples from PF/CO mice were used for interassay variation. The intraassay and interassay coefficients of variation were 6.1% and 5.8%, respectively.

### Quantitative RT-PCR analysis

Total RNAs in liver or epididymal adipose tissue were isolated using TRIzol^®^ Reagent (Invitrogen), and reverse transcription was conducted using the TaqMan Reverse Transcription Reagents Kit (Life Technologies). The primers used for each gene are listed in Supplementary Table S4. The values were normalized to *β-actin*, and are presented as fold changes, with the values of PF/CO set to one.

### Immunoblot analysis

Immunoblotting was performed as previously described^30^. The primary antibodies are listed in Supplementary Table S5. Densitometric analysis of the bands was performed using Quantity One software (Bio-Rad Laboratories, Inc., Hercules, CA, USA). The values were normalized to β-ACTIN and are presented as fold changes, with setting the values of PF/CO set to one.

### Statistical analysis

All data were expressed as means ± SD. Two-way analysis of variance (ANOVA) was initially performed to identify the effects of fat source, alcohol, and their interaction using GraphPad Prism 5.0 software. When the interaction was significant, data were tested by one-way ANOVA followed by Tukey’s post hoc test. Differences were considered significant at *P* < 0.05.

## Additional Information

**How to cite this article**: Wang, M. *et al.* Dietary α-linolenic acid-rich flaxseed oil prevents against alcoholic hepatic steatosis *via* ameliorating lipid homeostasis at adipose tissue-liver axis in mice. *Sci. Rep.* **6**, 26826; doi: 10.1038/srep26826 (2016).

## Supplementary Material

Supplementary Information

## Figures and Tables

**Table 1:** Routine parameters of C57BL/6 mice pair-fed a control or an ethanol liquid diet with corn oil and flaxseed oil.

Measurements	PF/CO	AF/CO	PF/FO	AF/FO	Two-way ANOVA		
					Ethanol	Fat	Interaction
Body weight, g	23.1 ± 0.7	22.6 ± 1.5	24.5 ± 0.7	23.6 ± 1.3	0.15	<0.05	0.77
Liver weight, g	0.79 ± 0.07^b^	0.99 ± 0.08^a^	0.84 ± 0.06^b^	0.94 ± 0.04^a^	<0.001	0.89	<0.05
LW/BW, %	3.42 ± 0.24	4.33 ± 0.18	3.37 ± 0.30	3.93 ± 0.11	<0.001	<0.05	0.06
Epididymal WAT, g	0.38 ± 0.07	0.26 ± 0.03	0.40 ± 0.08	0.34 ± 0.08	<0.05	0.09	0.51
eWAT/BW, %	1.55 ± 0.28	1.10 ± 0.26	1.62 ± 0.28	1.42 ± 0.30	<0.05	0.09	0.25
Plasma parameters							
AST, U/L	22.9 ± 5.0^c^	73.6 ± 11.7^a^	19.4 ± 5.1^c^	47.2 ± 14.1^b^	<0.001	<0.001	<0.01
ALT, U/L	14.9 ± 4.3^c^	50.8 ± 15.1^a^	11.8 ± 3.9^c^	28.7 ± 5.0^b^	<0.001	<0.05	<0.01
Glucose, mmol/L	8.03 ± 1.72	4.57 ± 1.17	8.25 ± 2.08	5.32 ± 1.65	<0.001	0.48	0.70
Insulin, ng/mL	0.70 ± 0.12	0.67 ± 0.11	0.71 ± 0.13	0.76 ± 0.26	0.94	0.56	0.66
Ethanol, nmol/L	0.87 ± 0.23	8.83 ± 1.72	1.04 ± 0.28	8.19 ± 1.54	<0.001	0.76	0.50
Adiponectin, μg/mL	39.5 ± 3.8	27.6 ± 4.3	40.4 ± 9.5	36.2 ± 5.5	<0.01	0.06	0.13

Values are expressed as the means ± SD (n = 8–10), and labeled means in a row without a common letter differ, *P* < 0.05; AF/CO, alcohol-fed with corn oil; AF/FO, alcohol-fed with flaxseed oil; ALT, alanine aminotransferase; AST, aspartate aminotransferase; BW, body weight; eWAT, epididymal white adipose tissue; LW, liver weight; PF/CO, pair-fed with corn oil; PF/FO, pair-fed with flaxseed oil.

**Table 2:** Fatty acid composition (%) of liver tissues obtained from C57BL/6 mice pair-fed a control or an ethanol liquid diet with corn oil and flaxseed oil.

Fatty acids			% of total fatty acids			Two-way ANOVA		
Common name	Symbol	PF/CO	AF/CO	PF/FO	AF/FO	Ethanol	Fat	Interaction
Myristic acid	14:0	0.40 ± 0.15	0.51 ± 0.22	0.41 ± 0.07	0.30 ± 0.06	0.84	<0.05	0.06
Pentadecanoic acid	15:0	0.11 ± 0.01	0.11 ± 0.02	0.12 ± 0.01	0.09 ± 0.01	0.02	0.40	<0.05
Palmitic acid	16:0	22.2 ± 1.0^a^	20.1 ± 3.8^b^	21.3 ± 0.8^a,b^	15.2 ± 1.6^c^	<0.001	<0.05	<0.05
Palmitoleic acid	16:1 n-7	1.61 ± 0.18^a^	0.86 ± 0.23^b^	1.38 ± 0.27^a^	0.98 ± 0.24^b^	<0.001	0.19	<0.05
Heptadecanoic acid	17:0	0.26 ± 0.03^b^	0.36 ± 0.03^a^	0.28 ± 0.04^b^	0.30 ± 0.04^b^	<0.001	0.19	<0.01
Heptadecenoic acid	17:1	0.16 ± 0.02	0.15 ± 0.02	0.22 ± 0.04	0.21 ± 0.05	0.20	<0.001	0.80
Stearic acid	18:0	10.8 ± 1.1^b^	16.5 ± 4.6^a^	16.5 ± 1.2^a^	15.8 ± 2.4^a^	<0.05	<0.05	<0.05
Oleic acid	18:1 n-9	15.9 ± 1.5	13.2 ± 2.9	12.8 ± 1.1	13.7 ± 2.4	0.28	<0.05	<0.01
Linoleic acid	18:2 n-6	23.2 ± 1.2	26.5 ± 4.4	13.3 ± 1.0	16.1 ± 1.0	<0.01	<0.001	0.80
γ- Linolenic acid	18:3 n-6	0.51 ± 0.04	0.65 ± 0.27	0.20 ± 0.02	0.24 ± 0.04	0.11	<0.001	0.33
α-Linolenic acid	18:3 n-3	0.28 ± 0.04^c^	0.47 ± 0.11^c^	8.30 ± 1.33^b^	14.5 ± 3.8^a^	<0.001	<0.001	<0.001
Arachidic acid	20:0	0.27 ± 0.04^b^	0.38 ± 0.18^a,b^	0.45 ± 0.05^a^	0.35 ± 0.07^a,b^	0.92	0.08	<0.05
Eicosenoic acid	20:1 n-9	0.56 ± 0. 10	0.38 ± 0.10	0.44 ± 0.11	0.37 ± 0.12	<0.01	0.09	0.12
Eicosadienoic acid	20:2 n-6	0.50 ± 0.09^b^	1.09 ± 0.74^a^	0.27 ± 0.04^b^	0.26 ± 0.05^b^	0.05	<0.01	<0.05
Dihomo-γ-linolenic acid	20:3 n-6	1.63 ± 0.13	1.07 ± 0.37	1.09 ± 0.16	0.82 ± 0.24	<0.001	<0.001	0.11
Arachidonic acid	20:4 n-6	12.6 ± 1.5^a^	9.20 ± 2.92^b^	4.40 ± 0.52^c^	4.77 ± 0.70^c^	<0.05	<0.001	<0.01
Eicosapentaenoic acid	20:5 n-3	0.14 ± 0.03^c^	0.17 ± 0.06^c^	8.16 ± 0.59^a^	5.86 ± 0.60^b^	<0.001	<0.001	<0.001
Behenic acid	22:0	0.36 ± 0.09	0.23 ± 0.09	0.51 ± 0.07	0.31 ± 0.08	<0.001	<0.001	0.30
Erucic acid	22:1 n-9	0.13 ± 0.05	0.12 ± 0.05	0.16 ± 0.03	0.14 ± 0.07	0.56	0.17	0.87
Docosatetraenoic acid	22:4 n-6	0.54 ± 0.09	0.49 ± 0.25	0.13 ± 0.04	0.11 ± 0.05	0.47	<0.001	0.75
Docosapentaenoic acid	22:5 n-3	0.29 ± 0.05	0.32 ± 0.08	1.89 ± 0.27	1.92 ± 0.27	0.66	<0.001	0.99
Lignoceric acid	24:0	0.22 ± 0.11	0.22 ± 0.06	0.29 ± 0.05	0.23 ± 0.07	0.20	0.15	0.31
Docosahexaenoic acid	22:6 n-3	6.29 ± 0.96	6.76 ± 2.05	7.63 ± 0.59	7.17 ± 0.87	0.99	0.07	0.34
Nervonic acid	24:1 n-9	0.20 ± 0.08	0.17 ± 0.05	0.27 ± 0.08	0.21 ± 0.06	<0.05	<0.05	0.52
SFAs		34.6 ± 1.3	38.4 ± 8.9	39.8 ± 1.4	32.6 ± 4.0	0.34	0.87	<0.01
MUFAs		18.1 ± 1.4	14.9 ± 3.4	14.7 ± 2.0	15.6 ± 2.6	0.07	<0.05	<0.01
n-3 PUFAs		7.01 ± 0.96	7.72 ± 2.31	26.0 ± 1.0	28.4 ± 3.0	<0.05	<0.001	0.09
n-6 PUFAs		39.0 ± 1.6	39.0 ± 8.99	19.4 ± 1.4	21.8 ± 2.1	0.21	<0.001	0.18
Total PUFAs		46.0 ± 2.1	46.7 ± 11.3	45.4 ± 1.6	51.3 ± 3.8	<0.05	0.13	0.05
n-6 PUFAs /n-3 PUFAs		5.66 ± 0.78	5.37 ± 1.46	0.75 ± 0.06	0.75 ± 0.12	0.68	<0.001	0.62

Values are expressed as the means ± SD (n = 8–10), labeled means in a row without a common letter differ, *P* < 0.05. AF/CO, alcohol-fed with corn oil; AF/FO, alcohol-fed with flaxseed oil; PF/CO, pair-fed with corn oil; PF/FO, pair-fed with flaxseed oil. SFAs value was calculated as (14:0 + 15:0 + 16:0 + 17:0 + 18:0 + 20:0 + 22:0 + 24:0); MUFAs value was calculated as (16:1 + 17:1 + 18:1 +  20:1 + 22:1 +  24:1); n-6 PUFAs value was calculated as (18:2 n-6 + 18:3 n-6 + 20:2 n-6 +  20:3 n-6 +  20:4 n-6 +  22:4 n-6); n-3 PUFAs value was calculated as (18:3 n-3 + 20:5 n-3 + 22:5 n-3 + 22:6 n-3).
