# Supplementary material for: Dietary α-linolenic acid-rich flaxseed oil prevents against alcoholic hepatic steatosis via ameliorating lipid homeostasis at adipose tissue-liver axis in mice
Source: Sci Rep. 2016 May 25;6:26826. doi: 10.1038/srep26826 (PMC4879538; doi:10.1038/srep26826)
Supplement: Supplementary Information [file srep26826-s1.pdf]

Dietary α-linolenic acid-rich flaxseed oil prevents against alcoholic hepatic steatosis *via* ameliorating lipid homeostasis at adipose tissue-liver axis in mice

Meng Wang[a], Xiao-Jing Zhang[a], Kun Feng[b], Chengwei He[a], Peng Li[a], Yuan-Jia Hu[a], Huanxing Su[a], Jian-Bo Wan[a,*]

[a] State Key Laboratory of Quality Research in Chinese Medicine, Institute of Chinese Medical Sciences, University of Macau, Macao

[b] Department of Bioengineering, Zunyi Medical College, Zhuhai Campus, Zhuhai, Guangdong, China

*Corresponding author

Dr. Jian-Bo Wan,

Room 6034, Building N22, Avenida da Universidade

University of Macau, Taipa, Macao.

Tel: +853-8822 4680;

E-mail: jbwan@umac.mo

Fatty acids		% of total fatty acids				Two-way ANOVA		
**Common name**	**Symbol**	**PF/CO**	**AF/CO**	**PF/FO**	**AF/FO**	**Ethanol**	**Fat**	**Interaction**
Myristic acid	14:0	0.99 ±0.06	1.15 ±0.21	1.09 ±0.19	0.88 ±0.07	0.73	0.23	<0.05
Palmitic acid	16:0	19.1 ±0.5	20.0 ±0.9	17.8 ±1.9	17.1 ±1.0	0.83	<0.05	0.17
Palmitoleic acid	16:1 n-7	4.77 ±0.82	3.41 ±1.19	4.26 ±0.99	3.02 ±0.49	<0.05	0.30	0.88
Stearic acid	18:0	2.51 ±0.39	3.98 ±0.94	2.93 ±0.48	4.59 ±1.10	<0.05	0.24	0.83
Oleic acid	18:1 n-9	31.8 ±0.9[a]	30.0 ±1.1[a, b]	28.4 ±2.7[b]	27.5 ±1.0[b]	0.14	<0.001	0.05
Linoleic acid	18:2 n-6	38.0 ±0.7	38.8 ±1.7	26.9 ±0.7	26.7 ±1.7	0.67	<0.001	0.43
α-Linolenic acid	18:3 n-3	1.61 ±0.31	1.52 ±0.50	19.6 ±2.3	19.5 ±2.1	0.90	<0.001	1.00
Dihomo-γ-linolenic acid	20:3 n-6	0.24 ±0.02	0.27 ±0.02	0.16 ±0.02	0.16 ±0.01	<0.05	<0.001	0.15
Arachidonic acid	20:4 n-6	0.44 ±0.08	0.45 ±0.05	0.21 ±0.09	0.20±0.03	0.91	<0.001	0.68
Eicosapentaenoic acid	20:5 n-3	0.09 ±0.03	0.08 ±0.02	0.23 ±0.12	0.19 ±0.03	0.33	<0.05	0.65
Docosapentaenoic acid	22:5 n-3	0.04 ±0.01	0.04 ±0.01	0.13 ±0.04	0.10 ±0.05	0.55	<0.05	0.55
Docosahexaenoic acid	22:6 n-3	0.15 ±0.02	0.22±0.05	0.21 ±0.09	0.17 ±0.04	0.55	0.10	0.09
SFAs		22.6 ±0.9	25.1 ±1.8	22.4 ±3.2	22.6 ±1.9	0.19	0.18	0.25
MUFAs		36.6 ±0.9[a]	33.4 ±0.6[b]	31.2 ±1.1[c]	30.5 ±1.2[c]	<0.05	<0.001	<0.05
n-3 PUFAs		2.16 ±0.65	1.90 ±0.56	20.1 ±2.4	19.9 ±2.0	0.77	<0.001	0.98
n-6 PUFAs		38.7 ±0.7	39.6 ±1.7	27.2 ±0.8	27.0 ±1.8	0.61	<0.001	0.43
Total PUFAs		40.8 ±1.2	41.5 ±1.4	46.4 ±2.2	46.9 ±1.9	0.50	<0.001	0.93
n-6 PUFAs /n-3 PUFAs		16.6 ±3.0	19.6 ±5.2	1.43 ±0.12	1.38 ±0.21	0.32	<0.001	0.31

Supplementary Table S1. Fatty acid composition (%) of adipose tissues obtained from C57BL/6 mice pair-fed a control or an ethanol liquid diet with corn oil and flaxseed oil

Values are expressed as means ± SD (n=4~6). Labeled means in a row without a common letter differ, $P < 0.05$. AF/CO, alcohol-fed with corn oil;

AF/FO, alcohol-fed with flaxseed oil; PF/CO, pair-fed with corn oil; PF/FO, pair-fed with flaxseed oil. SFAs value was calculated from (14:0 + 16:0 + 18:0); MUFAs value was calculated from (16:1 + 18:1); n-6 PUFAs was calculated from (18:2 n-6 + 20:3 n-6 + 20:4 n-6); n-3 PUFAs was calculated from (18:3 n-3 + 20:5 n-3 + 22:5 n-3 + 22:6 n-3); n-6 PUFAs /n-3 PUFAs means the ratio of n-6 PUFA to n-3 PUFA.

**Ingredients**	**PF/CO**	**AF/CO**	**PF/FO**	**AF/FO**
Casein, g/L	41.4	41.4	41.4	41.4
L-cystine, g/L	0.50	0.50	0.50	0.50
D,L-Methionine, g/L	0.30	0.30	0.30	0.30
Cellulose, g/L	10.0	10.0	10.0	10.0
Maltose Dextrin, g/L	115	44.8	115	44.8
Corn Oil, g/L	39.6	39.6	-	-
Flaxseed Oil, g/L	-	-	39.6	39.6
Mineral Mix (AIN93G-MX), g/L	8.8	8.8	8.8	8.8
Vitamin Mix (AIN-93-VX), g/L	2.5	2.5	2.5	2.5
Choline Bitartrate, g/L	0.53	0.53	0.53	0.53
Vitamin E Acetate, g/L	0.20	0.20	0.20	0.20
95% Ethanol (*v*/*v*), mL/L	-	52.6	-	52.6

Supplementary Table S2. Compositions of the modified Lieber-DeCarli liquid diets.

AF/CO, alcohol-fed with corn oil; AF/FO, alcohol-fed with flaxseed oil; PF/CO, pair-fed with corn oil; PF/FO, pair-fed with flaxseed oil.

Fatty acids		% of total fatty acids	
**Common name**	**Symbol**	**Corn Oil**	**Flaxseed Oil**
Lauric acid	12:0	0.2	0.2
Myristic acid	14:0	0.9	0.7
Palmitic acid	16:0	16.8	8.6
Palmitoleic acid	16:1 n-7	0.4	0.3
Stearic acid	18:0	4.1	5.4
Oleic acid	18:1 n-9	25.7	20.2
Linoleic acid (LA)	18:2 n-6	51.0	14.3
α-Linolenicacid(ALA)	18:3 n-3	1.0	50.3
SFAs		21.8	14.9
MUFAs		26.1	20.4
Total PUFAs		52.0	64.7
n-6 PUFAs/n-3 PUFAs		50.0	0.3

Supplementary Table S3. Fatty acid composition (%) of dietary fats contained in liquid diets.

The relative content of each fatty acid was quantified by normalization of the peak areas as the percentages of total fatty acids; MUFAs, monounsaturated fatty acids; SFAs, saturated fatty acids; PUFAs, polyunsaturated fatty acids. n-6 PUFAs/n-3 PUFAs means the ratio of n-6 PUFAs to n-3 PUFAs.

Gene	Full name	GeneBank accession No.	Primer sequences (forward/reverse)
Cd36	Fatty acid translocase	NM_001159556	5’-ATGGGCTGTGATCGGAACTG-3’<br>5’-TTTGCCACGTCATCTGGGTTT-3’
Fatp1	Fatty acid transport protein 1	NM_011977	5’-CGCTTTCTGCGTATCGTCTG-3’<br>5’-GATGCACGGGATCGTGTCT-3’
Lpl	Lipoprotein lipase	NM_008509	5’-TTGCCCTAAGGACCCCTGAA-3’<br>5’-TTGAAGTGGCAGTTAGACACAG-3’
Vldl-r	Very low-density lipoprotein receptor	MMU06670	5’-GGCAGCAGGCAATCGAATG-3’<br>5’-GGGCTCGTCACTCCAGTCT-3’
Fatp2	Fatty acid transport protein 2	NM_011978	5’-TCCTCCAAGATGTGCGGTACT-3’<br>5’-TAGGTGAGCGTCTCGTCTCG-3’
Fatp5	Fatty acid transport protein 5	NM_009512	5’-TCTATGGCCTAAAGTTCAGGCG-3’<br>5’-CTTGCCGCTCTAAAGCATCC-3’
Gpat	Glycerol-3-phosphate acyltransferase	NM_008149	5’-ACAGTTGGCACAATAGACGTTT-3’<br>5’-CCTTCCATTTCAGTGTTGCAGA-3’
Dgat1	Diacylglycerol O-acyltransferase 1	NM_010046	5’-TCCGTCCAGGGTGGTAGTG-3’<br>5’-TGAACAAAGAATCTTGCAGACGA-3’
Dgat2	Diacylglycerol O-acyltransferase 2	NM_026384	5’-GCGCTACTTCCGAGACTACTT-3’<br>5’-GGGCCTTATGCCAGGAAACT-3’
β-Actin	Beta-actin	NM_007393	5’-GGCTGTATTCCCCTCCATCG-3’<br>5’-CCAGTTGGTAACAATGCCATGT-3’

Supplementary Table S4. A list of genes and their corresponding primers used in the quantitative RT-PCR analysis.

Primary antibody	Full name	Molecular Weight	Maker
ATGL	Adipose triglyceride lipase	54 kDa	Cell Signaling
p-HSL(Ser^{660})	Phospho-hormone-sensitive lipase	81 kDa	Cell Signaling
HSL	Hormone-sensitive lipase	81 kDa	Cell Signaling
p-IRE1α (Ser^{724})	Phospho-inositol-requiring enzyme 1 alpha	110 kDa	Abcam
IRE1α	Inositol-requiring enzyme 1 alpha	110 kDa	Abcam
p-EIF2α (Ser^{51})	Phospho-eukaryotic translation initiation factor 2 subunit alpha	38 kDa	Bioworld Technology
EIF2α	Eukaryotic translation initiation factor 2 subunit alpha	38 kDa	Bioworld Technology
PPARG	Peroxisome proliferator-activated receptor- gamma	53,57 kDa	Cell Signaling
CD36	Fatty acid translocase	88 kDa	Santa Cruz Biotechnology
FATP5	Fatty acid transport protein 5	75 kDa	Santa Cruz Biotechnology
MTTP	Microsomal triglyceride transfer protein	97 kDa	Santa Cruz Biotechnology
ADIPOR2	Adiponectin receptor 2	44 kDa	Santa Cruz Biotechnology
p-AMPK (Thr^{172})	Phospho-AMP-activated protein kinase	62 kDa	Cell Signaling
AMPK	AMP-activated protein kinase	62 kDa	Cell Signaling
p-ACC (Ser^{79})	Phospho-acetyl-CoA carboxylase	280 kDa	Cell Signaling
ACC	acetyl-CoA carboxylase	280 kDa	Cell Signaling
β-ACTIN	BETA-ACTIN	42 kDa	Cell Signaling

Supplementary Table S5. Primary antibodies used in the immunoblot analysis.
